# Changes of right-hemispheric activation after constraint-induced, intensive language action therapy in chronic aphasia: fMRI evidence from auditory semantic processing^1^

**DOI:** 10.3389/fnhum.2014.00919

**Published:** 2014-11-14

**Authors:** Bettina Mohr, Stephanie Difrancesco, Karen Harrington, Samuel Evans, Friedemann Pulvermüller

**Affiliations:** ^1^Department of Psychiatry, Charité Universitätsmedizin, Campus Benjamin FranklinBerlin, Germany; ^2^Department of Psychology, Anglia Ruskin UniversityCambridge, UK; ^3^Institute of Cognitive Neuroscience, University College LondonLondon, UK; ^4^Medical Research Council, Cognition and Brain Sciences UnitCambridge, UK; ^5^Brain Language Laboratory, Department of Philosophy and Humanities, Freie Universität BerlinBerlin, Germany

**Keywords:** fMRI, aphasia, constraint-induced aphasia therapy, intensive language action therapy, brain reorganization, stroke, language

## Abstract

The role of the two hemispheres in the neurorehabilitation of language is still under dispute. This study explored the changes in language-evoked brain activation over a 2-week treatment interval with intensive *constraint induced aphasia therapy* (CIAT), which is also called *intensive language action therapy* (ILAT). Functional magnetic resonance imaging (fMRI) was used to assess brain activation in perilesional left hemispheric and in homotopic right hemispheric areas during passive listening to high and low-ambiguity sentences and non-speech control stimuli in chronic non-fluent aphasia patients. All patients demonstrated significant clinical improvements of language functions after therapy. In an event-related fMRI experiment, a significant increase of BOLD signal was manifest in right inferior frontal and temporal areas. This activation increase was stronger for highly ambiguous sentences than for unambiguous ones. These results suggest that the known language improvements brought about by intensive constraint-induced language action therapy at least in part relies on circuits within the right-hemispheric homologs of left-perisylvian language areas, which are most strongly activated in the processing of semantically complex language.

## Introduction

A highly disputed issue in cortical reorganization and language recovery after post stroke aphasia (PSA) is the role of the right hemisphere (RH) as opposed to perilesional brain regions in the left language-dominant hemisphere (LH). The classic perspective had been that language is a function of the left hemisphere in almost all right-handed individuals, and, in this spirit, some current approaches to aphasia therapy even aim at suppressing right hemispheric activity in areas homotopic to left-hemispheric language regions. Consistent with the primary role of the LH in language, a majority of studies report evidence of language reorganization in perilesional areas within the LH in PSA patients (Heiss et al., 1999; Price and Crinion, 2005; Breier et al., 2007; Meinzer et al., 2008). However, it may be that recovery of function supported by preserved language pathways in perilesional regions is limited to patients with small LH lesions. In contrast, patients with large LH lesions, including damage in an extended area of the perisylvian language network, must recruit different neuronal structures to establish new language networks potentially including homotopic regions of the RH.

A range of findings suggest that the RH makes important contributions to language processing in both the healthy and lesioned brain and, more specifically, in patients with fluent as well as non-fluent aphasia (Basso et al., 1989; Basso and Macis, 2011). In particular, RH areas homotopic to the left-hemispheric inferior frontal and temporal language areas seem to be recruited in well recovered aphasic patients (Weiller et al., 1995). Activation dynamics in the RH may therefore reflect the success of neurorehabilitation of language functions (Musso et al., 1999; Saur et al., 2006). There is evidence that at least some of these new language substrates in patients with large lesions may be located in the non-dominant RH (Cappa and Vallar, 1992; Crosson et al., 2005; Heiss and Thiel, 2006; Turkeltaub et al., 2012). Apart from etiology, lesion size and site, time post lesion and other variables, language recovery, and cortical reorganization in aphasia seems to be influenced significantly by pre-morbid language use and therapeutic intervention (Crosson et al., 2005; Marcotte and Ansaldo, 2010).

To date, only very few functional neuroimaging studies have investigated the effects of successful aphasia therapy on brain reorganization. Hence, the underlying neuronal processes that may lead to effective language re-learning and cortical re-organization after stroke are still largely unclear. When assessing the neuronal changes that accompany therapeutic intervention, it is of prime relevance to exclude any changes in functional brain organization that could be attributed to spontaneous recovery rather than to aphasia therapy. Therefore, it is advantageous to focus on chronic patients (>1 year post stroke) for whom spontaneous recovery of neural function is very unlikely (Berthier and Pulvermüller, 2011). Furthermore, the type of therapy, along with the intensity and outcome of the treatment may have a great influence on neuroplasticity. Therefore, it seems most promising to assess the effects of aphasia therapy on brain reorganization by choosing a therapeutic method that has been demonstrated to be effective at the chronic stage and yields its beneficial effect within a relatively short period of time. Finally, an appropriate choice of the assessment task is essential. When studying functional language activation before and after therapy, it is important to use a task which covers important aspects of language use and can be performed effectively by aphasic patients. In this context, it has been highlighted that structural properties of the patient's native language play an important role, since the same underlying deficit may have different formal manifestations in different languages (Paradis, 2001).

Intensive Language Action therapy (ILAT), an intensive form of aphasia therapy, also called Constraint-Induced Aphasia Therapy (CIAT) (Pulvermüller et al., 2001; Pulvermüller and Berthier, 2008), has proven to be successful in patients with chronic aphasia (Bhogal et al., 2003; Kelly et al., 2010; Berthier and Pulvermüller, 2011). ILAT is a 2 weeks intensive therapy programme typically applied with 3 h of training per day. In has been developed on the basis of established knowledge from the neurosciences and involves practicing language skills that are modeled on spoken communication in everyday situations (Pulvermüller and Berthier, 2008). In randomized controlled clinical trials, ILAT has been shown to be effective in improving language functions after only 10 consecutive weekday sessions in patients with chronic post-stroke aphasia (Pulvermüller et al., 2001; Meinzer et al., 2005; Berthier and Pulvermüller, 2011). Therapeutic benefits could even be demonstrated in long-term follow-up studies, in chronic patients who often had not received any language therapy for years or even decades prior to the therapy (Meinzer et al., 2005; Berthier and Pulvermüller, 2011).

Brain activation studies have provided somewhat diverging results with regard to the brain manifestations of language reorganization. Pre-therapy RH activation has been shown to be a predictor of immediate clinical success post-therapy, but may not predict the stability of language improvements over time or indeed be linked to post-therapy changes in RH activation (Breier et al., 2006; Richter et al., 2008). Instead, ILAT-induced functional language improvement seems to be associated with re-recruitment of intact LH regions in the vicinity of the lesion (Meinzer et al., 2008; Breier et al., 2009). In spite of occasional reports of bihemispheric language reorganization in post-stroke aphasia (Pulvermüller et al., 2005; Breier et al., 2006; Kurland et al., 2012), these findings seemingly challenge the evidence reviewed previously for a prime role of homotopic RH language regions in aphasia recovery (e.g., Weiller et al., 1995).

Functional recovery of language at the chronic stage of post-stroke aphasia is due to neuroplasticity and in particular to synaptic plasticity driven by Hebbian learning. In theory, therapy can lead to the formation of novel language circuits but it is likely that much of the restoration of language functions seen in chronic aphasia is due to the “repair”—that is, strengthening and possibly extension—of pre-existing language circuits functionally impaired by focal cortical lesion. As, in the healthy cortex, neuronal circuits processing words and meaningful constructions are distributed over both hemispheres (Mohr et al., 1994) and even reach into sensory and motor areas (Kiefer and Pulvermüller, 2012), the frequent concurrent activation of linguistic forms in the context of, and constrained by, relevant communicative actions and perceptions may bring about efficient strengthening of such partly lesioned networks (for a broader discussion of neuroplasticity due to CIAT/ILAT, please see Berthier and Pulvermüller, 2011). On this background and because areas in the still intact non-dominant RH are especially important for semantic processing (Zaidel, 1976), it appears straightforward to expect reorganization of semantic networks to be particularly prominent in the RH. Any right-hemispheric reorganization may best be revealed by tasks requiring in-depth processing of word and sentence meaning. This reasoning led us to apply a semantic task with different levels of semantic processing load to monitor neuroplasticity changes induced by ILAT.

In the present study, an auditory sentence comprehension task was used to investigate patterns of brain activation before and after 2 weeks of ILAT in a group of chronic aphasic patients. In this task, sentences were presented containing either two semantically-ambiguous words, or matched control sentences without ambiguities. For example, the word “bark” can either refer to the rough surface of a tree or the noise made by a dog, and hence additional processes of semantic selection and combination are required to comprehend sentences containing ambiguous words compared to match low-ambiguity sentences consisting of words without obvious ambiguities. Because a degree of ambiguity cannot be excluded for most sentences—as they can be meant in minimally different “senses” and referring to different entities—we will use the terms “high-“ and “low-ambiguity conditions.” Previous studies have demonstrated additional activation for high-ambiguity sentences in bilateral inferior frontal gyros (IFG) and left posterior inferior temporal lobe (Zempleni et al., 2007). An auditory semantic ambiguity task has also been applied in brain injured (minimally-conscious and vegetative) patients with intact speech comprehension and elicit the same brain activation patterns for semantically ambiguous and unambiguous sentences (Coleman et al., 2007). This finding shows that the ambiguity task leads to reliable neurometabolic signals in severely brain-injured patients, thus making it a promising tool for application in stroke patients. In addition to the contrast of high- and low-ambiguity sentences, an additional non-speech control condition was included using signal correlated noise (SCN). In healthy and brain-injured volunteers alike the contrast of sentences vs. non-speech noises was found to lead to activation in anterior and posterior portions of the superior and middle temporal gyrus (Rodd et al., 2005; Coleman et al., 2007), including the superior-temporal regions involved in processing speech sounds (Rauschecker, 1999; Price et al., 2005; Rauschecker and Scott, 2009).

In addition to using semantic ambiguity as a neural marker for higher-level speech comprehension, we also included a behavioral assessment of the patients' abilities to comprehend high-ambiguity sentences. Typically, brain-damaged patients with lesions in left perisylvian regions show impairments in language understanding (Bates et al., 2003), including semantic processing and resolving lexical ambiguities. When processing short sentences containing ambiguous words, two patients with damage specifically to the left IFG, but who presented with no residual comprehension problems, were slower at resolving lexical ambiguities compared to both healthy controls and a patient with non-left IFG damage (Vuong and Martin, 2011). Furthermore, results from previous studies show that patients with non-fluent aphasia resulting from LH damage were impaired at selecting the contextually appropriate meanings of ambiguous words, such that appropriate and inappropriate meanings were chosen equally often following a constraining sentence (Grindrod and Baum, 2003). These findings suggest that the LH and the left IFG in particular are critical in processing lexical ambiguities. We therefore anticipated that aphasic patients with LH lesions might demonstrate difficulties in a semantic ambiguity task and further that effective language therapy could lead to improvements in semantic processing of ambiguous words in sentences, combined with neuronal changes in these respective areas. Whether LH perisylvian processes or rather RH homotopic ones, or both, contribute to the reorganization of ambiguity resolution remains to be investigated.

The present study therefore employed three measures to assess therapy-related changes: (i) clinical language assessment, (ii) a behavioral auditory semantic ambiguity task, and (iii) a functional magnetic resonance imaging (fMRI) task with auditory presentation of high and low ambiguity sentences. Clinical, behavioral and neuronal changes were measured after a 2 weeks intensive intervention programme with ILAT. It was hypothesized that chronic aphasic patients would show improvements of language functions after therapy. In addition, we expected to find changes in BOLD activation in response to highly ambiguous sentences, relative to low-ambiguity materials, either in perilesional areas of the inferior-frontal and temporal core language cortex in the LH, or in homotopic RH regions, or in both.

## Methods

### Participants

Twelve patients (5 females, mean age: 54.5 years, range: 26–76 years) with chronic non-fluent aphasia were tested immediately before and after participating in a 2 weeks language therapy interval with ILAT (Difrancesco et al., 2012). All participants presented with mild to moderate language impairments and were recruited from self-help groups.

Patients were selected according to the following criteria: (i) language impairment following a single stroke affecting the territory of the left middle cerebral artery (one patient suffered from hemorrhage, all others from ischemic strokes), assessed by structural MRI scans. (ii) monolingual native speakers of English before stroke, assessed by a language history questionnaire (none of the patients had been fluent in any language other than English at any time before stroke). (iii) right-handedness before stroke. Handedness was assessed with the Edinburgh Handedness Inventory (Oldfield, 1971). (iv) no psychiatric diagnosis. Patients with severe comprehension deficits who were not able to fully engage in therapy were also excluded.

Four patients were not able to undergo fMRI testing due to health risks. Data from a further two patients could not be included in the pre-post analysis due to technical problems during the pre-scanning sessions and consequent data loss. The final pre-post fMRI analysis therefore included six right-handed patients (1 female, aged 41–76). Table 1 provides clinical and demographic data for each of the eight patients who underwent fMRI scanning, from which the two patients with incomplete MRI data were excluded. Language was assessed with the naming test (Boston Naming Test, BNT) from Boston Diagnostic Aphasia Battery (BDAE, Goodglass and Kaplan, 1972), including its part on naming (BNT) and language comprehension, and the Token Test (TT, De Renzi and Vignolo, 1962). Scores from subtests of the BDAE and the TT before and after the therapeutic intervention are presented in Table 2.

**Table 1:** **Neurological and demographic data of eight patients who underwent fMRI scanning**.

**P**	**Age (in years, at therapy)**	**Sex**	**Handedness**	**Duration of aphasia (months)**	**Etiology**	**Lesion site**
1	74	Male	Right	127	Ischemia	Left MCA territory, extending from left frontal to left occipital and parietal area
2	69	Male	Right	25	Ischemia	Left MCA territory, in LIFG, with additional small lesion in left IPC
3	48	Male	Right	17	Ischemia	Left MCA territory, extending from LIFG to STG
4	72	Male	Right	32	Ischemia	Left MCA territory, extending from left frontal to posterior STG and parietal area
5	60	Female	Right	137	Ischemia	Left MCA territory, extending from left frontal to left temporal and IPC
6	41	Male	Right	19	Ischemia	Left SFG extending posteriorly to left IPC, with additional lesion in left ITG
7	76	Male	Right	234	Hemorrhage	Small lesion in posterior LIFG, extending to insula
8	59	Male	Right	104	Ischemia	Left MCA territory, extending from LIFG to left temporal and IPC
Average	63.38			88.88		

*MCA, middle cerebral artery; LIFG, left inferior frontal gyrus; SFG, superior frontal gyrus; IPC, inferior parietal cortex; STG, superior temporal gyrus; ITG, inferior temporal gyrus*.

**Table 2:** **Clinical assessment of language functions administered before and after ILAT**.

	**BDAE**		**BDAE**				**TT**	
	**Auditory comprehension**		**Syntactic processing**		**BNT**		**(Number of errors)**	
**Patient**	**Before**	**After**	**Before**	**After**	**Before**	**After**	**Before**	**After**
1	85	86	22	20	12	23	17	14
2	81	85	15	21	13	19	31	33
3	85	87	11	12	12	24	41	35
4	81	84	16	9	36	32	48	38
5	83	80	18	16	26	27	15	18
6	80	81	16	14	8	14	49	38
7	88	90	21	22	48	48	16	17
8	82	86	15	15	48	51	35	30
Mean	83.13	84.88^*^	16.75	16.13	25.38	29.75^*^	31.50	27.88^*^
*SD*	2.70	3.23	3.54	4.58	16.69	13.31	14.16	9.96

*BDAE (Boston Diagnostic Aphasia Battery; Goodglass and Kaplan, 1972)—Auditory comprehension composite score includes word comprehension, word comprehension by categories, and semantic probe subtests; Syntactic processing composite score includes touching A with B, reversible possessives and embedded sentences subtests; BNT, Boston Naming Test. TT, Token Test (De Renzi and Vignolo, 1962)*.

* *Indicates significant differences between pre and post-therapy assessment*.

The study was approved by the Cambridgeshire Local NHS Research Ethics (NRES) Committee.

### Aphasia therapy

Patients participated for 2 weeks in ILAT, which was administered for 3–4 h per day for 10 consecutive week-days. Testing took place immediately before and after the therapy. Details of the therapeutic setting and regime can be found in Pulvermüller et al. (2001) and Difrancesco et al. (2012). Clinical and psycholinguistic testing and neuroimaging was done before and after therapy.

## Experiment 1: behavioral semantic ambiguity resolution task (pre-scanning)

In a behavioral experiment preceding fMRI scanning, patients were asked to engage in a semantic ambiguity task which lasted for approximately 10 min and was carried out in a quiet room before fMRI scanning. This was done to familiarize patients with the task and to monitor if patients were able to make semantic decisions above chance level.

### Materials

Thirty six high-ambiguity and 36 low-ambiguity sentences spoken by a native English female speaker were presented to the patients. High ambiguity sentences contained an ambiguous word (e.g., *“plant”*) which was disambiguated later on in the sentence toward the subordinate (less-frequent) meaning of the word (i.e., “*the student believed the plant should never have been built*”). Each high ambiguity sentence was matched to a low ambiguity sentence for syntactic structure, number of syllables, number of words, sentence duration, target and disambiguating word length, target word frequency and log frequency, disambiguating word frequency, and the number of senses for the disambiguating word. Low ambiguity sentences contained minimally ambiguous words (i.e., “*the students believed the hill could never have been climbed*”). The duration of the sentences ranged from 2.21 to 2.29 s.

For each sentence, two probe words were generated. Probe words for the high ambiguity sentences were either *related* to the contextually-appropriate meaning of the ambiguous word (i.e., *manufacture*) as disambiguated in the sentence or *irrelevant* (i.e., *tree*) a word that was related to the context-irrelevant, dominant meaning of the ambiguous word. Probe words for the low ambiguity sentences were a *related probe*, which was related to the sentence meaning, and an *unrelated probe* not related to the sentence meaning. Probe words were matched across conditions for length, frequency, and log frequency.

### Procedure

All stimuli were presented using E-prime Professional 2.0 (Psychology Software Tools, Pittsburgh). A cross in the center of the screen indicated the beginning of a trial. Patients were instructed to press any key on a button box to begin a trial when they were ready. Each sentence was presented auditorily one at a time, followed by a 2000 ms pause. Patients were instructed to listen carefully to the sentences. After the presentation of each sentence, two probe words were visually presented on a computer screen. One word was presented to the left of fixation and the second word was presented to the right of fixation. Both words stayed on the screen until patients had made their responses. Patients were instructed to decide which of the two words best fit with the meaning of the sentence and to press one of two buttons which corresponded to the side of the screen on which the related probe word was presented. In addition, probe words were presented auditorily. This was done to help patients with reading problems who had difficulties to read the visually presented words, as multisensory presentation of stimuli seems to enhance cognitive processing (Diederich and Colonius, 2004).

Patients were given three trials not included in the main task as a practice run.

The 36 items from each condition were split into two equal runs, each including 18 pairs of matched high- and low-ambiguity sentences. The two runs were also matched for number of syllables per sentence, duration, rated naturalness and dominance scores of the ambiguous word. Stimuli in the behavioral task were different from those used in the fMRI task. Patients engaged in the behavioral semantic ambiguity task before and after language therapy. The order of stimulus presentation was counterbalanced across the two testing sessions.

## Experiment 2: fMRI correlates of semantic ambiguity resolution

### Methods

#### Materials

Stimuli were taken from Rodd et al. (2005) and consisted of two auditory speech conditions (high-ambiguity and low-ambiguity sentences) and a low-level noise control condition. There were 57 stimulus items in each condition. The high ambiguity sentences contained two ambiguous words which were disambiguated in the sentence (e.g., “there were *dates* and *pears* in the fruit bowl”). Ambiguous words could be either homonyms (two meanings, but identical spelling and pronunciation, e.g., “bark”) or homophones (two meanings, different spelling, identical pronunciation, e.g., “knight”/“night”). The ambiguous words in all sentences had an inappropriate meaning that belonged to the same syntactic category as the correct interpretation, so that both meanings were possible considering the purely syntactic context. Therefore, disambiguation had to be done on the basis of semantic rather than syntactic information provided by the sentence context. Each sentence was matched to a low ambiguity sentence for number of words and syntactic structure. Low ambiguity words contained minimally ambiguous words (i.e., “there was beer and cider on the kitchen shelf”). The physical duration of the sentences ranged from 1.2 to 4.3 s and the two conditions were matched for number of syllables, physical duration, rated naturalness, imageability and mean frequency of the content words in the CELEX database (log-transformed word frequency, Baayen et al., 1995). Imageability and naturalness ratings were obtained by using a 9-point Likert scale (whereby the number 9 indicated highly imageable or natural stimuli). None of the psycholinguistic factors revealed any significant difference between the two sentence types (all *p*-values > 0.1). Table 3 presents descriptive statistics of stimulus characteristics. The 57 sentences in each condition were then divided into three sets of 19 sentences—one of these sets of sentences was selected for use in either the pre- and post-therapy scanning session. A further set of 19 sentences provided ambiguous words to be used in the control condition of the post-scanning word association task. Assignment of sentences to conditions was counterbalanced over patients.

**Table 3:** **Stimulus properties and ratings (means) for high and low ambiguity sentences used in the fMRI experiment**.

**Sentence type**	**Length in syllables**	**Length in words**	**Length in seconds**	**Naturalness**	**Imageability**	**Log word frequency**
High-ambiguity	11.6	9.3	2.2	6.3	5.2	4.5
Low ambiguity	11.8	9.3	2.2	6.4	5.3	4.7

*In none of the physical and psycholinguistic factors assessed did the two sentence groups differ significantly from each other. (all ps > 0.1)*.

Fifty seven sentences not used in either speech condition but matched for number of syllables, number of words and physical duration were converted to SCN. These items were derived from a white noise shaped to have the same long term average spectrum and amplitude envelope as the speech stimuli. They were unintelligible and contained no recognizable speech sounds, words or meanings, and sound like fluctuating static noise. SCN was used as a low-level baseline condition to allow assessment of activity in low-level auditory areas (in Heschl's gyrus) compared to rest, and to assess speech vs. non-speech activity in lateral temporal regions (Mummery et al., 1999). An additional 19 trials (in each scanning run) contained silent rest periods to allow for comparisons between low-level SCN activation and silent rest periods.

Sentences were spoken by a female native speaker of English. All stimuli were presented using E-prime Professional 2.0 (Psychology Software Tools, Pittsburgh). Patients were instructed to listen attentively to the sentences without performing an explicit task. Previous research has shown equivalent activation of inferior frontal and inferior temporal regions during semantic ambiguity resolution in active and passive listening conditions (Rodd et al., 2005).

#### fMRI procedure and data acquisition

A sparse imaging technique was used to minimize interference from scanner noise (Hall et al., 1999). Participants heard a single sentence, an SCN equivalent or silence in a 9 s period before a 2 s whole-brain EPI acquisition. The timing of stimulus onset was altered for each sentence or noise condition, in order that the midpoint of each stimulus was temporally aligned with a point 5 s before the midpoint of the subsequent scan (i.e., 5 s into the 9 s silent period). This ensured that the hemodynamic response to the sentence had reached an approximate maximum at the time of the subsequent scan.

The 57 trials for each condition were split into three sessions of 79 scans which included 19 items per condition (high/low-ambiguity/SCN) as well as 19 silence trials plus two dummy scans at the start and one dummy scan at the end of the scanning run. Items from each condition were equally distributed across the three scanning runs and pseudo-randomized to ensure that each condition occurred equally often after each of the other conditions. One of these three scanning runs was delivered at each of the two time points tested (i.e., pre- and post-therapy). The assignment of specific sentence materials to the pre- and post-therapy scanning runs was counterbalanced over patients.

All images were acquired on a Siemens 3 T Tim Trio scanner (Siemens Medical Solutions, Camberley, UK) with a 32-channel head coil. Echo planar image volumes were acquired during a scanning session lasting approximately 12 min. Each scanning volume consisted of 32 slices, each 3 mm thick, with an interslice gap of 0.75 mm and in-plane resolution of 3 × 3 mm, *FOV* = 192 × 192 mm; Total *TR* = 11 s (*TE* = 30 ms, acquisition time = 2 s). A T1-weighted structural image was acquired for each patient using an MPRAGE sequence (*TR* = 2250 ms, *TE* = 2.99 ms, flip angle = 9°, *FOV* = 256 × 240 × 160 mm, voxel size = 1 × 1 ×1 mm).

#### fMRI analysis

FMRI data were pre-processed and analyzed using Statistical Parametric Mapping software (SPM 8, Wellcome Department of Imaging Neuroscience, London, UK). Pre-processing steps included within-subject realignment, spatial normalization of the functional images to a standard EPI template and spatial smoothing using a Gaussian kernel of 10 mm. Data were analyzed using the General Linear Model (GLM) modeled in scans using the canonical HRF filter. At the first level the pre and post scanning sessions were modeled within the same design matrix. Within each participant's first level model, each condition was modeled separately, in addition to the scanning session mean, and six movement regressors of no interest. Second level whole brain analysis was conducted for SCN vs. Rest. This was done by taking images from the first level up to the second level to conduct random effects one sample t-tests. Regions of interest (ROIs) were defined using Marsbar (Brett et al., 2002) and orthogonal contrasts from this study, or from previously published findings in healthy volunteers. For each patient, activation values within each ROI were averaged for each scanning session and the significance of neural differences between pre and post therapy scanning sessions was assessed using the MarsBar software. In addition small volume corrections (SVC, Worsley et al., 1996) were also conducted within localized ROI. For this contrast, SVCs were generated by examining activity within regions activated in healthy volunteers for [Speech > SCN] whilst excluding lesioned tissue on an individual participant basis (Seghier et al., 2008). These search volumes were localized in the LH and RH, centered on the superior temporal sulci, extending to anterior and posterior middle temporal gyri and additionally included a portion in the inferior frontal gyri, bilaterally.

As mentioned before, for two patients, issues with the timing of stimulus presentation as controlled by E-prime precluded statistical analysis of fMRI data. Therefore, only data from the six remaining patients who successfully completed pre- and post-scanning sessions, was further analyzed.

#### Lesion identification analysis

**Figure 1:**
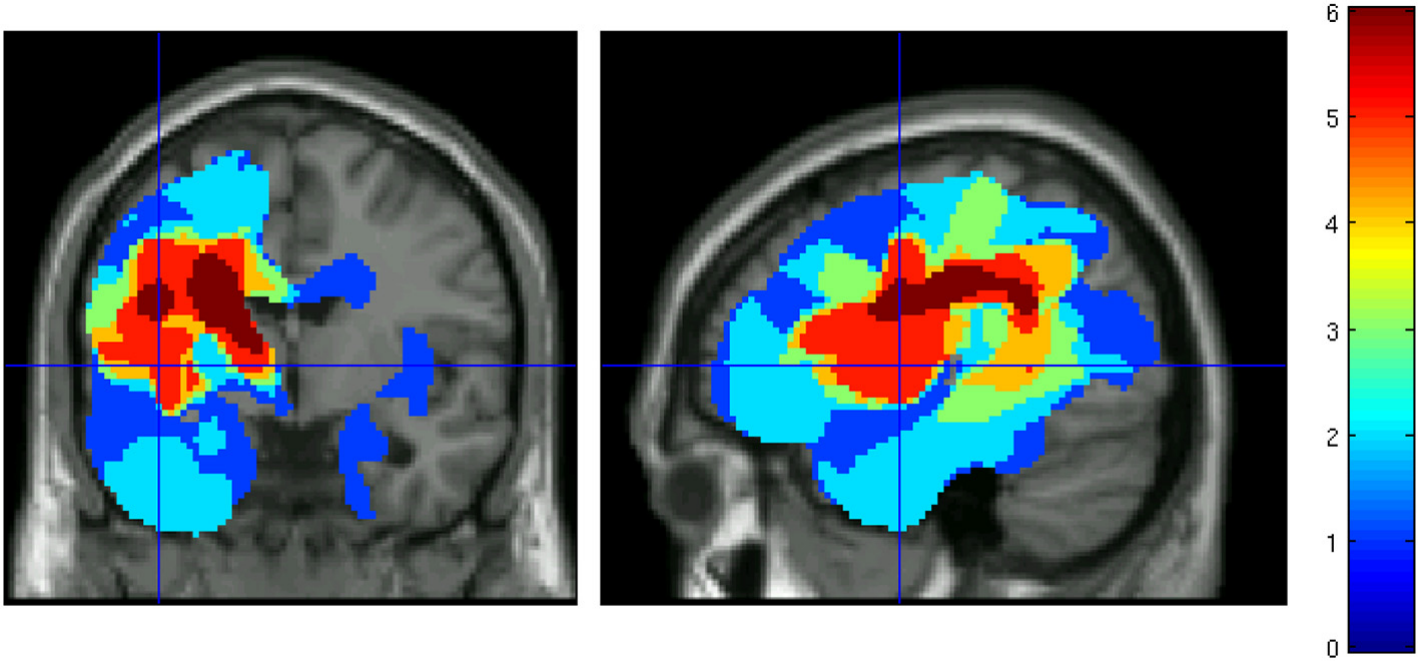
**Lesion overlap map for patients who successfully underwent fMRI scanning before and after aphasia therapy**. Color indicates the number of patients with damage at each voxel.

To obtain a lesion map identifying the sites of major damage in each patient's brain, damaged tissue was identified using the Automatic Lesion Identification (ALI) toolbox (Seghier et al., 2008). This method involves augmenting the standard generative model for unified segmentation (as implemented in SPM8) with an empirical prior for a “lesioned tissue class” that is optimized iteratively. Healthy gray and white matter tissue maps were established by segmenting structural brain images of a group of age matched neuro-typical individuals (60 individuals, mean = 61, min = 43, max = 75 years) taken from a wider reported corpus (Peelle, 2012). These healthy gray and white matter segmentations, following normalization and smoothing, were used as the basis for establishing outlier gray and white matter in the patient group (i.e., lesioned tissue) using a fuzzy clustering procedure. Individual binary lesion images were combined to create a lesion probability map, showing the spatial extent and anatomical overlap of lesions for the group of patients (see Figure 1). Across the patients, damage covered a number of key left-hemisphere language regions around and extending from the Sylvian fissure, including the inferior and middle frontal gyrus, inferior and superior temporal gyrus (STG), inferior parietal areas, hippocampus, and left insula. The damage also extended to underlying white matter, following the curve of the arcuate fasciculus.

For subsequent analyses, individual participant ROIs were defined based on brain regions activated for high and low ambiguity sentences in a previous study of healthy volunteers (Rodd et al., 2005), while excluding lesioned tissue. Four main ROIs were defined for the ambiguity contrast: left inferior frontal, left temporal, right inferior frontal and right temporal. RH ROIs were generated by mirroring the left hemisphere activation observed in healthy volunteers. ROIs for the contrast of sentences and non-speech noises were also taken from previous data collected in healthy volunteers included a large region of the superior and middle temporal gyri, extending both anterior and posterior along the superior temporal sulcus and additionally included a portion of the inferior frontal gyri. Bilateral and symmetrical ROIs were generated by mirroring and combining the (typically bilateral) activation seen for speech vs. non-speech contrasts in healthy volunteers.

### Results

#### Standardized clinical language tests (pre-post therapy)

**Figure 2:**
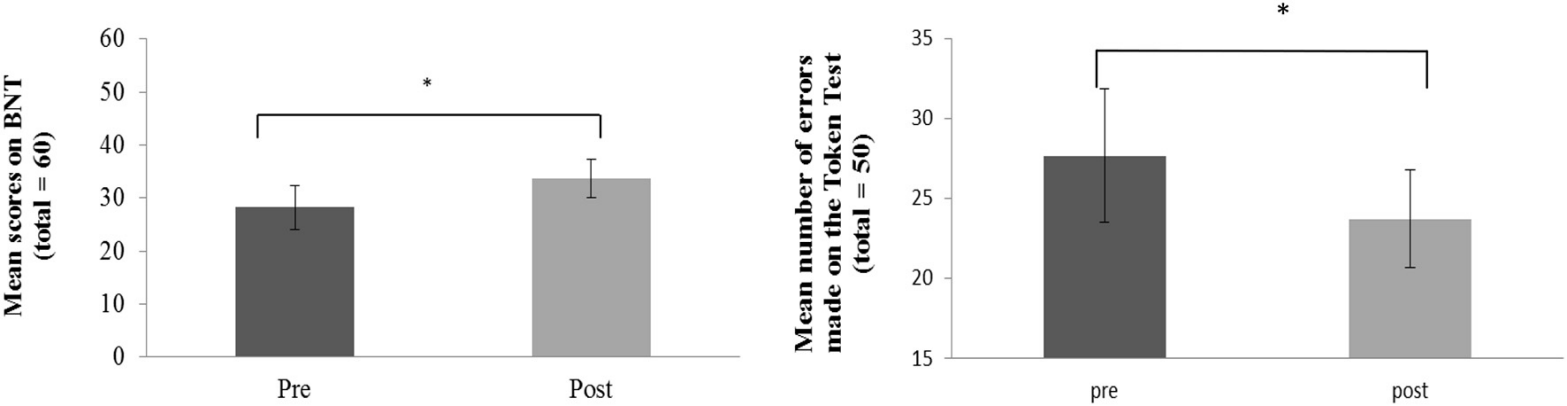
**Behavioral improvement over therapy in the patient group: Average pre and post therapy scores for number of correct items named on the Boston Naming Test (left panel) and number of errors on the Token Test (right panel)**. Error bars represent standard errors. Asterisks indicate significance level at *p* < 0.05.

Clinical language assessment pre and post therapy was analyzed using paired samples t-tests. Results showed significant improvements in language functions after the 10-day treatment interval in the BNT, *t*_(7)_ = 2.26, *p* = 0.029; in the *Auditory Comprehension* subtest of the BDAE *t*_(7)_ = 2.67, *p* = 0.032 and in the TT *t*_(7)_ = 2.20, *p* = 0.048 (which assesses patients' auditory language comprehension abilities). Significant improvements in the BNT and TT after therapy are displayed in Figure 2.

In addition, a normalized change score was calculated over all three language tests (BNT, TT, auditory comprehension) taken together to assess general clinically manifest language performance. Results demonstrated significant improvements of overall language performance after therapy *t*_(7)_ = 2.67, *p* = 0.016. These results were largely confirmed by additional analyses performed for the six patients whose data entered the pre-post fMRI analysis. A significant improvement in the *BNT*, *t*_(5)_ = 1.20, *p* = 0.024, a marginally significant improvement in the *Auditory Comprehension* test *t*_(5)_ = 1.58, *p* = 0.087 and again a significant improvement of overall language performance were evident *t*_(5)_ = 2.02, *p* = 0.049.

## Experiment 1: behavioral assessment of semantic ambiguity resolution (pre-and post-therapy)

**Figure 3:**
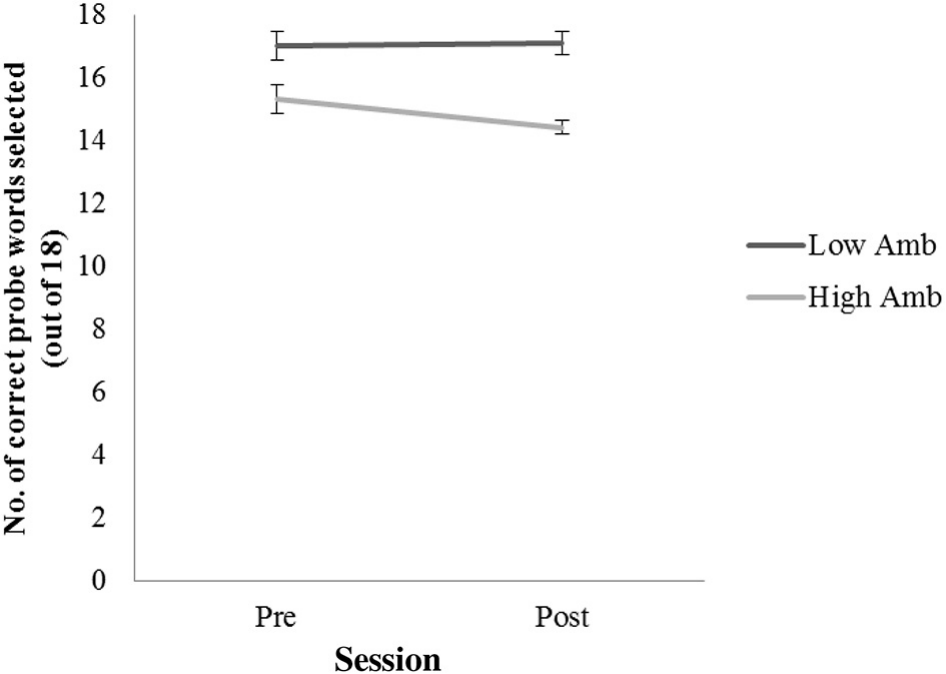
**Number of correct probe words selected for low ambiguity (low Amb) and high ambiguity (High Amb) sentences across two time sessions (pre and post therapy)**. Error bars represent standard error.

A two-factor repeated measures ANOVA assessed accuracy in selecting sentence-appropriate probe words for high-and low ambiguity sentences pre-and post-therapy. Patients were significantly more accurate at selecting the correct probe word for low- compared to high-ambiguity sentences [*F*_(1,14)_ = 36.149, *p* < 0.001]. These data are displayed in Figure 3. There was no difference in the proportion of correct answers across the two testing sessions, neither was there an interaction between the factors session (pre and post therapy) and sentence type (high and low ambiguity). One-sampled t-tests demonstrated that patients correctly chose the relevant probe words for high ambiguity sentences significantly more often than at chance level at both the pre- [*t*_(7)_ = 14.04, *p* < 0.001] and post-therapy [*t*_(7)_ = 22.42, *p* < 0.001] testing sessions. This suggests that patients were able to extract the correct meaning from both high and low ambiguity sentences and make appropriate semantic ambiguity decisions. This pattern remained the same after the 2 weeks of therapy.

## Experiment 2: fMRI correlates of auditory and speech perception and semantic ambiguity resolution

### fMRI: signal-correlated-noise vs. silence

#### Individual analyses of whole brain activity

Acoustic processing of auditory stimuli was assessed by comparing responses from SCN (i.e., non-speech) to a silence period where no stimulation took place. This contrast identified brain regions involved in acoustic processing which are unrelated to higher level speech processing. In healthy participants, SCN stimulation compared to rest led to activation of the primary auditory cortex, surrounding Heschl's gyrus (Davis and Johnsrude, 2003). As expected, this contrast comparing BOLD responses elicited by SCN with silence, showed anatomically appropriate responses in Heschl's gyrus in all patients, similar to healthy controls, both during pre- and post-therapy sessions. This activation survived correction for multiple comparisons at the whole brain level (*p* < 0.05 Family Wise Error correction, FWE). There was no evidence for any change of this pattern of activation over therapy.

### fMRI: speech vs. signal-correlated-noise

Language-specific brain activation was investigated by comparing the BOLD responses averaged over high and low ambiguity sentences with the SCN condition. This speech vs. non-speech contrast allowed identification of brain regions specifically involved in auditory language processing.

**Figure 4:**
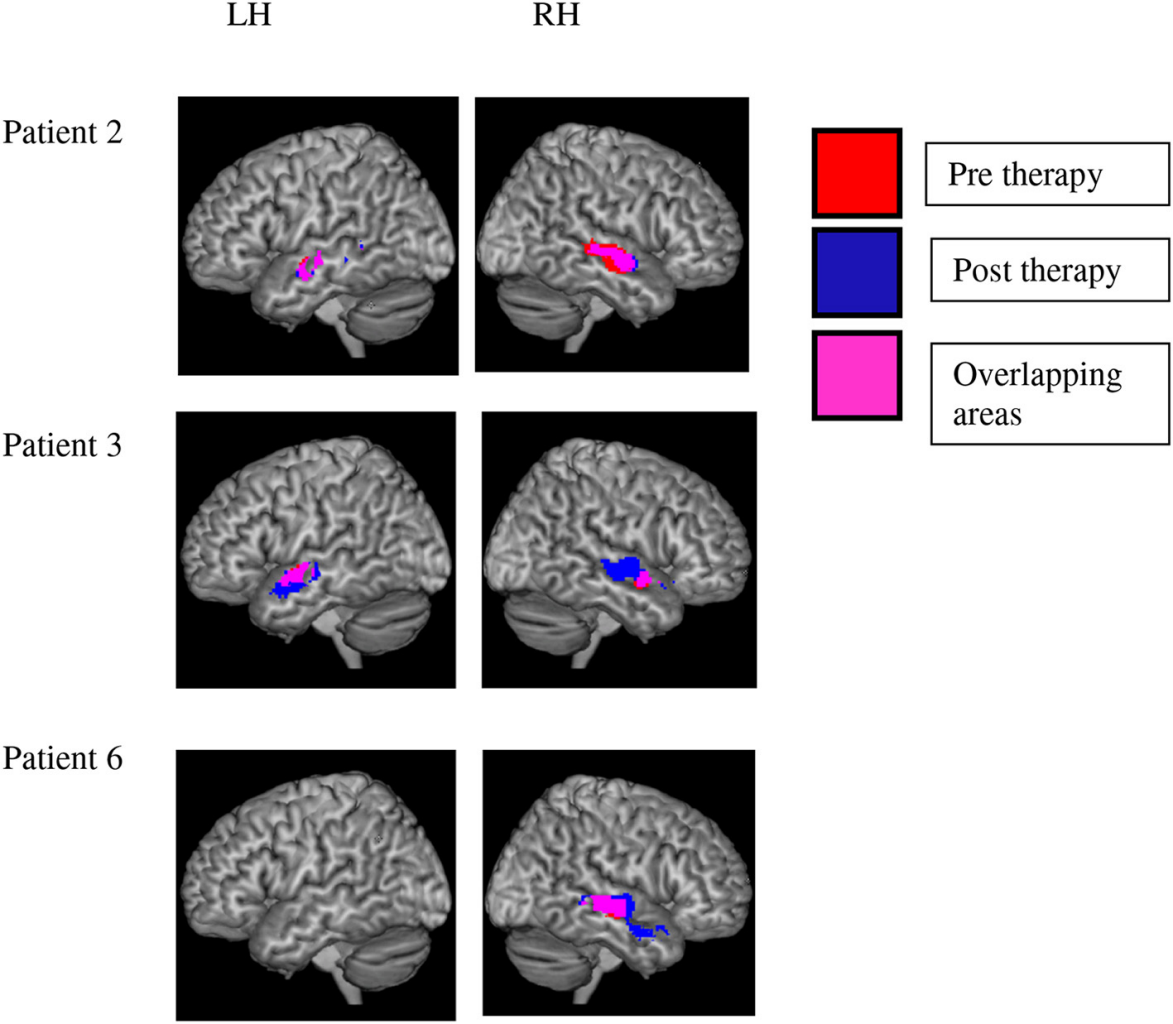
**Variability of brain activation to speech as compared with signal-correlated-noise, SCN**. Significant activation to speech vs. SCN after small volume correction is displayed in the left and right hemisphere of three patients. Red voxels indicate activation before and blue ones after the therapy interval. Pink voxels show significant activation before and after therapy. Statistical analyses failed to reveal any significant change of activation in the speech vs. SCN contrast over therapy in any of the patients. Note the variability of speech-related activation over patients.

The [speech > SCN] contrast revealed activation in the temporal lobe which survived significance after SVC in three patients. Significant results were found at pre and post-therapy testing sessions, although direct pre-post comparisons were nonsignificant (see below). Two of the patients (2 and 3) showed significant bilateral activation in middle and STG, extending to the temporal poles. One patient (patient 6) showed extensive activation in the left hemisphere only, in middle temporal gyrus extending to the middle temporal pole. These results are evidence of substantial variability in the localization of brain processes that distinguish between speech and noise in aphasic patients (see Figure 4).

There were no significant activation clusters for the pre-post [speech > SCN] comparison for any of the patients individually. In addition, none of the voxels that showed stronger activation to speech than noise gave evidence of significant changes of the speech-noise contrast over therapy (pre vs. post contrast). Thus, our results failed to support the hypothesis that speech processing in general was altered by therapy—as compared with the noise baseline.

A group analysis showed speech vs. SCN activation at a low threshold (*p* < 0.001, uncorrected) in both hemispheres before and after therapy, which, however, did not survive SVC. This non-significance after correction matches the observation of variability in the precise regions involved in this contrast, which emerged from the single subject analyses. Again no evidence for a change over therapy of speech-evoked activations in general was found relative to SCN.

### fMRI: high ambiguity vs. low ambiguity sentences

Higher-level processing of semantic information was addressed by comparing BOLD responses to high ambiguity vs. low ambiguity sentences. This contrast allowed us to identify additional neural activity specifically associated with highly complex semantic processes when patients had to process sentences containing semantically ambiguous as opposed to unambiguous words, for example: pears/pairs or night/knight. In healthy volunteers, this contrast produced activation in the left posterior inferior temporal lobe as well as in left IFG (Rodd et al., 2005). Activation in these areas in patients would therefore provide a neural correlate for higher level semantic processing of sentences consistent with the successful ambiguity resolution shown by the behavioral data.

**Figure 5:**
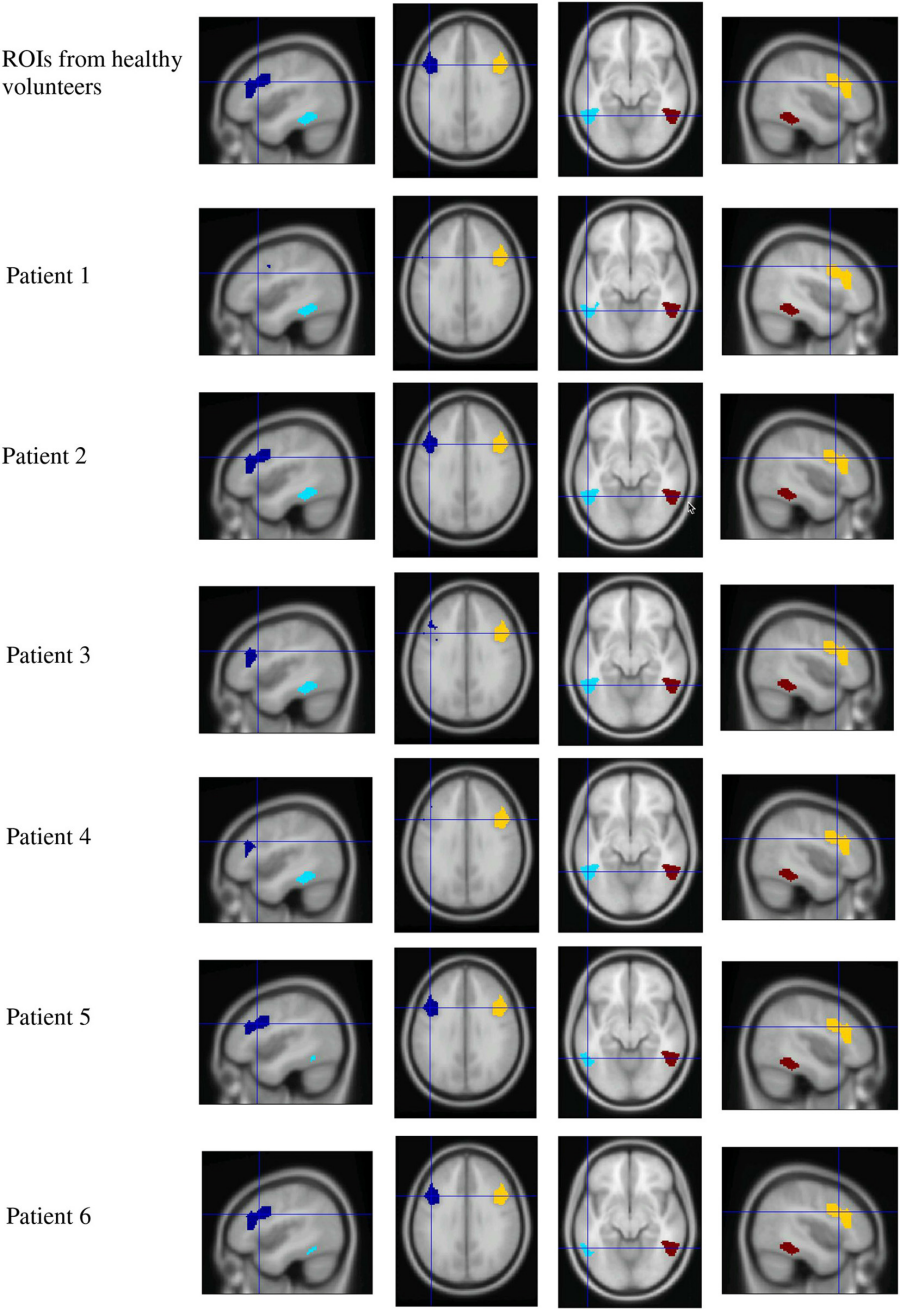
**Region of interest maps for the high vs. low ambiguity contrast**. Four ROIs from healthy volunteers were defined by contrasting high ambiguity sentences > low ambiguity sentences (taken from Rodd et al., 2005, upper row). Lesioned tissue within each individual patient was excluded from ROIs, resulting in reduced extension of LH ROIs in patients. (Dark blue, left frontal ROI; light blue, left temporal ROI; yellow, right frontal ROI; red, right temporal ROI).

A Marsbar *ROI analysis* was conducted. ROIs were defined by using the contrast [High > Low Ambiguity], which had been reported to be significant at the group level in healthy volunteers (Experiment 2 of Rodd et al., 2005). MNI coordinates for selected ROI centers were for a*mbiguous vs. unambiguous sentences*: LH IFG (−49, 28, 18); RH IFG (44, 14, 30); LH STG (−52, −51, −7.4); RH STG (52, 51, 7). Figure 5 shows ROIs for each individual patient (after projection and removal of lesioned tissue) used in the ambiguity contrast analysis.

**Figure 6:**
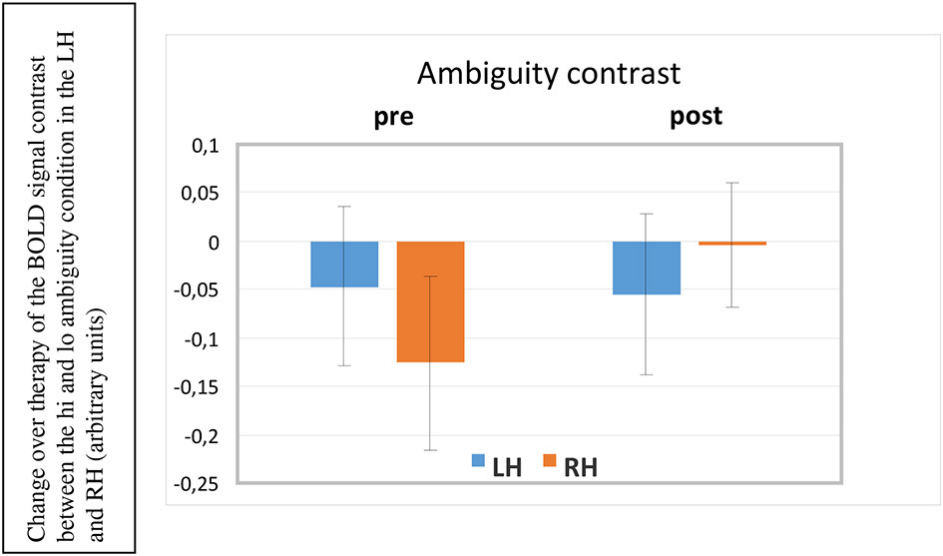
**BOLD ambiguity contrast (high vs. low ambiguity sentences) in the left and right hemisphere (LH, RH) before and after therapy (pre, post)**. As the relative activation is plotted, negative bars mean that unambiguous sentences led to stronger brain activation than the more complex ambiguous ones. This was the case before therapy in the right hemisphere. After therapy, this RH under-activation to semantically ambiguous sentences disappeared (significant interaction of the factors Prepost × Hemisphere, *p* < 0.034).

A 2 × 2 × 2 (ROI × Hemisphere × Session) repeated measures ANOVA documented a significant two-way interaction between the factors scanning *Session* and *Hemisphere* [*F*_(1, 5)_ = 8.349, *p* = 0.034] revealing a significant change in activation in the RH in the post-therapy session compared to the pre-treatment session, but no change in the left hemisphere across the two sessions (see Figure 6). Before therapy, right hemispheric activation was significantly stronger for unambiguous sentences than for ambiguous ones, but after therapy, this difference had disappeared. Please recall that healthy controls had shown stronger activation for ambiguous sentences than for unambiguous ones in these same ROIs; the reverse effect in our patients was therefore surprising. In this context the vanishing of unambiguous sentence-superiority in the RH of aphasic patients over therapy can be seen as a step toward typical brain activation. We will discuss this result in more detail below.

## Discussion

The present study investigated the neuronal changes underlying language recovery consequent to ILAT in a group of stroke patients with chronic aphasia. ILAT was administered during a period of 10 consecutive days. Before and after intervention, patients underwent clinical language assessment, behavioral testing and fMRI scanning whilst listening to ambiguous and unambiguous sentences and matched non-speech sounds. The results revealed that (i) ILAT led to significant improvements of language functions over a short period of time, (ii) patients were able to successfully perform lexical ambiguity decisions, and, (iii) neurometabolic changes over therapy in the processing of semantically more or less complex sentences occurred in inferior-frontal and superior-temporal areas of the RH adjacent to regions homotopic to the perisylvian language areas in the left hemisphere. After therapy, relative neurometabolic activation increase was manifest in a task requiring in-depth linguistic-semantic analysis of otherwise well-matched language materials, so that the neural dynamics may be attributed to brain mechanisms of semantic processing of ambiguous sentences.

The present results confirm previous findings that ILAT, an aphasia therapy based on neuroscientific principles, can lead to recovery of language functions and to changes in cortical activation patterns within a very short period of time (Pulvermüller et al., 2001; Meinzer et al., 2008; Kurland et al., 2012). Previous findings are extended by demonstrating therapy-related neurometabolic changes related to semantic ambiguity processing. In contrast with some previous reports (Meinzer et al., 2008) and in agreement with others (Pulvermüller et al., 2005; Richter et al., 2008), our data suggest a prime role of the RH in functional reorganization of language after intensive speech therapy, especially in the domain of semantic language processing. Significant clinical improvements were found in patients' naming abilities and sentence comprehension, measured by the BNT, the Auditory Comprehension subtest and the TT, respectively. Due to the careful selection of chronic patients (>1 year post stroke), it seems unlikely that the observed language improvements can be attributed to spontaneous recovery. Interestingly, although naming is not explicitly practiced in ILAT, improvements in naming abilities after ILAT have also been found in previous studies (Pulvermüller et al., 2001; Meinzer et al., 2005; Maher et al., 2006; Berthier et al., 2009; Difrancesco et al., 2012). Moreover, improvements of performance in the TT after ILAT have been reported previously (Pulvermüller et al., 2001) and reflect improvements in receptive (semantic and syntactic) language abilities due to successful training with ILAT. In this context, it should be noted that ILAT focuses on communicative speech acts such as “making a request” or “planning an action.” Improvements in specific language abilities such as naming therefore constitutes evidence for a generalization of therapy effects over speech acts (from requesting to naming).

Why did right hemispheric circuits, but not left hemispheric ones, reveal neuroplastic changes in the current study? Building on the suggestions offered in the introduction, we wish to hypothesize that, in the healthy individual, semantic circuits for words and constructions are spread out over both hemispheres and that lesion in left-perisylvian language areas renders these circuits functionally deficient. If aphasia therapy with high frequency leads to strengthening of synaptic connections in the pre-existing but damaged circuits, and, in addition, putatively, to incorporation of new neurons into the cell assemblies, the perisylvian areas of the RH homotopic to the language regions of the left are prime candidates for such re-organization of language mechanisms. Such reorganization is consistent with the improved performance of our patients on the BNT and their overall clinical language performance. As the RH is especially important for the reorganization of semantic circuits, it is not surprising that metabolic changes—the relative enhancement of BOLD signals seen in the inferior-frontal and superior-temporal ROIs on the right—are clearly apparent in a demanding semantic condition, where stimulus words and constructions offered different semantic understandings (high ambiguity condition). In theory, such reorganization should also be apparent in a low load semantic condition, but given the low power of our present study with only six patients, one cannot expect to statistically confirm such an effect. In future, larger numbers of patients need to be scanned to replicate the present result of signs of most pronounced reorganization in the right non-dominant hemisphere for in-depth semantic processing, and to investigate the possibility of similar (but milder) effects in simple semantic tasks. It should also be assessed whether aphasic patients have any specific impairment in ambiguity processing, which reduces in the course of aphasia therapy. Our present post-scanning sentence judgment task was aimed to confirm understanding of sentences by all patients and successfully revealed high near-ceiling performance, which did not document a behavioral correlate of improvements in ambiguity processing. On the other hand, this absence of an effect is important for interpreting the present results, as a lack of ambiguity understanding in our patients before therapy would forbid any strong interpretation of the neurometabolic changes in terms of semantic processing. In this case, subjects could simply have not listened to some of the ambiguous items leading to reduced brain activation in the pre-therapy session. As good performance was seen before and after therapy, the fMRI changes can be interpreted as signs of a change in the processes supporting successful semantic processing.

The main aspect of this study was to determine cortical reorganization as a result of language training with ILAT. In order to map brain areas that might show changes in neuronal activation during the course of the therapy, a semantic ambiguity task seemed ideal, as it had produced activation patterns in language-relevant brain regions (Rodd et al., 2005), was manageable by aphasic patients, challenging enough to prevent performance at ceiling levels and resembles natural language use in everyday life, which always serves the communication of meaning at different levels of complexity.

Behavioral data from the semantic ambiguity task performed by patients before scanning revealed that semantic ambiguity decisions were well above chance (average: 88.9%) and yielded high accuracy scores, similar, but below those obtained in healthy controls (average: 97%). These results fit with previous reports that aphasic patients are able to use sentence contexts for disambiguating semantically ambiguous words (Vuong and Martin, 2011). In contrast to healthy controls, who did not show differences between high and low ambiguity decisions (Rodd et al., 2005), patients were better at making decisions for low ambiguity sentences, indicating that high ambiguity sentences might have been slightly more challenging for patients than low ambiguity sentences. Importantly, however, there were no significant changes on this semantic task when pre- and post-therapy performance was compared.

The main rationale for employing a behavioral task in addition to the fMRI task was to obtain a behavioral measure of the patients' ability to distinguish words with different levels of semantic ambiguity. This ensured that patients were able to reliably process stimuli presented in the scanner, as the fMRI task did not include a behavioral component. It should be noted that there was no difference in behavioral measures of semantic ambiguity decision before and after the therapy, as patients showed similar accuracies in pre- and post-therapy testing sessions. This may be due to the fact that patients already performed at ceiling level at the pre-therapy testing session (see Figure 3). This view is supported by the low standard errors, indicating that all patients were able to manage the task with very few errors, both before and after ILAT. However, given that some patients had rather large lesions affecting most parts of the fronto-temporal language network in the left hemisphere, it is surprising how well patients were able to retrieve the correct semantic meaning of the ambiguous words, which were comparable to accuracy levels of healthy individuals.

As the number of patients who could be included in the pre-post fMRI analysis was rather small, we performed analyses of individual data sets for each contrast, followed by group analyses. The paradigm employed here has already been successfully applied in single-subject analyses (Coleman et al., 2007) and our findings demonstrate neural activation which even reached significance at a conservative threshold corrected for multiple comparisons in some of the single-subject analyses. Some aspects of these activations were comparable to activation seen in a group of healthy volunteers while others seemed especially variable over patients.

The SCN condition was included to control for unspecific (non-speech) effects on the auditory system. For non-linguistic acoustic stimuli matched to our sentence materials (SCN), there was no consistent pattern in brain activation changes over therapy. It should be noted that all patients showed anatomically appropriate activation to auditory stimuli, comparable to that of healthy volunteers (Rodd et al., 2005).

When contrasting BOLD signals elicited by speech (both ambiguity conditions combined) with those to SCN, we found significant activation clusters in the RH in middle and STG, extending to the temporal pole. These contrasts survived SVC in three out of the six patients (Figure 4), thus demonstrating a robust effect in these individuals. Whereas right hemispheric activation was significant—with some local variation in all three patients, only two showed additional left-hemispheric SVC-significance for the contrast speech vs. SCN, while healthy controls activate a substantial portion of their perisylvian fronto-temporal cortex for this contrast. In this context, our present findings suggest that this contrast may be less pronounced in patients and variable over individuals, even in its degree of laterality between the two hemispheres. For interpreting our remaining results, it is important to stress that the speech vs. non-speech contrast failed to give evidence of any *changes over therapy*. None of the single subject or group analyses performed on this contrast revealed significant activation increase or decrease between the pre- and post-sessions. The significant change seen in the ambiguity contrast is therefore a unique feature of our present results.

The ambiguity contrast comparing BOLD responses to high ambiguity stimuli with those to low ambiguity stimuli showed a significant session by hemisphere interaction (*p* = 0.03), indicating that highly ambiguous sentences elicited significantly more activation, relative to low-ambiguity sentences in inferior frontal and temporal regions in the RH after the therapy compared with before, whereas left-hemispheric activation contrasts remained unchanged. No statistically reliable differences emerged between the frontal and temporal ROIs; therefore, only the laterality factor (hemisphere) can be interpreted. Before therapy, the right non-dominant hemisphere showed a relatively reduced activation to ambiguous sentences compared with unambiguous ones. This means that those right-hemispheric frontal and temporal areas that normally—in healthy individuals—respond more strongly to ambiguous than to unambiguous sentences, actually responded less in our patients. This finding suggests that the relative reduced activation to ambiguous sentences may indicate a processing problem for these difficult items. After therapy, the relative under-activation in the RH to ambiguous sentences had disappeared, now showing comparable activation patterns to unambiguous and ambiguous items, indicating less effortful processing for these complex stimuli. This change can be seen as one step toward normal language processing and may potentially indicate that patients engaged more strongly in processing semantically complex materials after treatment.

Contrasting with these right-hemispheric findings, there was no evidence for change in neuronal activation in the left hemisphere over time. In healthy volunteers, inferior frontal regions, and inferior temporal regions in both cortical hemispheres have previously been identified as crucial in resolving lexical ambiguities and therefore seem to be involved in the complex processing of semantic linguistic information (Rodd et al., 2005; Zempleni et al., 2007). These results further demonstrate the usefulness of semantically demanding tasks and stimuli in revealing neuroplastic changes related to language reorganization which may be difficult to reveal with more basic speech contrasts (for example, speech vs. SCN).

As functional language training emphasizing processing of meaning and the differentiation of semantic properties in a communicative context is a key feature of ILAT, the current dynamics of the brain correlates of semantic processing sit well with its application. However, any strong conclusions on specific effects of ILAT on semantic brain mechanisms must wait further testing in randomized controlled trials. In general, the observed changes in activation in semantic networks in right-hemispheric areas following 2 weeks of ILAT are consistent with the idea that massed practice of behaviorally relevant language may aid functional recovery after stroke (Taub et al., 2002; Berthier and Pulvermüller, 2011). The neurometabolic changes may have correlates in RH functional connectivity and could be due to increased activation in already established but strengthened language circuits or to formation of new circuits as a result of intensive language therapy.

The analysis of activation patterns in individual patients indicates that some patients showed an increase in activation in right frontal and temporal regions after therapy and that this activation increase was most pronounced for high ambiguity sentences. Already in healthy volunteers, bilateral frontal, and temporal activation enhancement was seen to ambiguous sentences compared with matched unambiguous ones (Rodd et al., 2005). It appears that it is especially the RH functional network that shows plasticity over ILAT. However, it should be noted, that this group result could not be confirmed by each patient at a single subject analysis level and it appeared that especially patients with strong activation in the LH failed to show evidence of any neurometabolic changes over the treatment period. Hence, while the overall group analysis revealed significant activation changes in frontal and temporal areas of the RH, individual data sets reveal a much more complex pattern of topographic changes over therapy. The degree of therapy-induced recovery from brain damage can be very heterogeneous as are variations in brain activation patterns, reported by previous studies (Meinzer et al., 2008). This can be attributed to different variables such as location and extent of brain lesion, responsiveness to language therapy, motivation, or even premorbid factors (Crosson et al., 2005). In addition, it should be noted that the lack of additional significant changes after therapy in the semantic ambiguity task (both, behaviorally and physiologically) could be attributed to the small sample size that was included in the pre-post analysis.

Changes in brain activation after ILAT have been reported previously and show mixed results with regards to lateralization of language after therapy. In our previous study, EEG and source localization before and after ILAT showed a word-specific enhancements of a negative going event related potential (latency 250 ms) generated in both hemispheres (Pulvermüller et al., 2005) when patients performed a lexical decision task. Both studies agree on the result that right-hemispheric activation increased over therapy with ILAT in chronic aphasic subjects, although the left-hemispheric activation increase could not be confirmed at the group level in the present study. Thus, our present fMRI data suggest a stronger role of the RH in cortical reorganization. However, it should be noted that the two studies differed in several aspects, including task, imaging method, stimulation modality and language used and are therefore difficult to compare in a direct fashion. EEG studies found that bilateral activation of superior temporal areas predict therapy outcome after ILAT (Pulvermüller et al., 2005), while long-term improvements of language functions after ILAT were associated with LH activation (Breier et al., 2009). Other studies employing fMRI have found ILAT-related changes of hemodynamic brain activation in perilesional areas of the LH (Kurland et al., 2012) as well as in homotopic areas of the RH (Saur et al., 2006). Similar to our results, these studies report a rather mixed pattern of therapy-induced activation changes in both hemispheres for individual patients. Despite the fact that a number of previous studies found evidence for perilesional in the LH accompanying functional recovery, it seems that the non-dominant RH plays a crucial role in the recovery of verbal communication after stroke in general and after ILAT in particular. However, the factors, influencing RH contribution to cortical reorganization still seem to be unresolved, although task and stimulus properties seem to play an important role. For example, in contrast to our findings, Richter et al. (2008) found that pre-therapy RH activation was followed by a decrease in activation after ILAT and correlated with clinical outcome and some researchers even argue that inhibiting right inferior-frontal areas in aphasic patients by repetitive TMS may improve language performance (Naeser et al., 2012). Our present results speak against such an interpretation as the orchestration of RH activation in different semantic contexts seemed to be the best indicator of and, potentially, an important substrate for the neuroplasticity of language.

As functional language changes were seen over a period of 2 weeks only, while none of the patients received any other speech and language therapy, the observed functional changes are likely to be a result of treatment. However, a major shortcoming of the present study is that no control group could be included to control for treatment specific effects or repetition effects. Therefore, it is not entirely clear from our data if the clinical and neurometabolic changes seen after the 2-week treatment interval are specifically related to the administration of ILAT, to language therapy in general or even just due to repetition effects. Therefore, a more controlled design or, ideally, a randomized controlled trial is required to assess this more clearly. It should be noted, though, that previous reports have shown stability in brain activation patterns across test-retest sessions in healthy controls within a 2-week interval and in patients with aphasia who underwent a baseline scan 3 weeks before the pre-therapy scan (Breier et al., 2009). Therefore, it seems rather unlikely that our data are a result of pure repetition effects. Although the present results must be considered with caution, they add to a growing body of evidence for ILAT related neural changes.

In conclusion, our data further confirm that ILAT is effective in treating patients with chronic aphasia. It could also be shown that aphasia patients are able to make semantic ambiguity decisions when ambiguous and unambiguous sentences were presented auditorily. Employing a semantic ambiguity paradigm may therefore provide a useful tool for assessing higher-level language processing and functional changes after aphasia therapy. The present findings indicate that 2 weeks of ILAT lead to a change in brain activity in inferior frontal and temporal areas of the right, unaffected hemisphere of chronic aphasia patients. This activation change occurred specifically while patients were processing sentences containing highly ambiguous words. The data support the notion that brain regions in the unaffected RH, homotopic to typical LH language areas, may contribute to functional reorganization in chronic aphasia.

### Conflict of interest statement

The authors declare that the research was conducted in the absence of any commercial or financial relationships that could be construed as a potential conflict of interest.
